# Neuroprotective Effects of the Ethanolic Leaf Extract of *Crassocephalum crepidioides* (Asteracaeae) on Diazepam-Induced Amnesia in Mice

**DOI:** 10.1155/2022/1919469

**Published:** 2022-09-28

**Authors:** Beppe Galba Jean, Folefack Alice Irène, Atsamo Albert Donatien, Ngatanko Aïbassou Hervé-Hervé, Barga Mpoo Bertrand, Allah-Doum Nanou Gael, Nguedia Ymele Merline, Zemo Gamo Franklik, Dongmo Alain Bertrand, Dimo Théophile

**Affiliations:** ^1^Department of Biological Science, Faculty of Science, University of Maroua, P.O. Box 814, Maroua, Cameroon; ^2^Department of Animal Biology and Physiology, Faculty of Science, University of Yaounde I, P.O. Box 812, Yaoundé, Cameroon; ^3^Department of Psychology, Faculty of Arts, Letters and Social Science, University of Yaoundé I, P.O. Box 7011, Yaoundé, Cameroon; ^4^Department of Animal Biology Faculty of Science University of Douala, P.O. Box 24157, Douala, Cameroon

## Abstract

This study aimed to evaluate the neuroprotective effects of the ethanolic leaf extract of *Crassocephalum crepidioides* (*Cc*) on diazepam-induced amnesia in mice. Thirty mice distributed into six groups of five mice each were used. The normal control and negative control groups received 2% ethanol per os, the positive control group received piracetam (150 mg/kg, *p.o*), and three experimental groups were treated with three doses of ethanolic leaf extract of *Cc* (100, 200, and 400 mg/kg, *p.o*). All groups except the normal control group were co-treated with diazepam (3 mg/kg, *i.p*) daily for 14 days. The memory effects were evaluated using the Radial Arm Maze (RAM) and the Novel Object Recognition (NOR) tests, while the anti-depressive effects were evaluated using the tail suspension test. All animals were sacrificed at the end of the study. Hippocampi, isolated from the right hemisphere, were used to prepare a homogenate for the determination of oxidative stress biomarkers. The ethanolic leaf extract of *cc* significantly (*p* < 0.001) decreased the number of working and reference memory errors in the RAM test and induced a significant (*p* < 0.01) increase in the time spent exploring the novel object in the NOR test. The extract also induced a significant (*p* < 0.001) increase in the mobility time in tail suspension. Moreover, compared to the negative control group, the extract significantly (*p* < 0.01) increased superoxide dismutase activity and significantly (*p* < 0.01) decreased malondialdehyde levels. The histopathological analysis of hippocampi showed that the *cc* extract increased cell density when compared with the negative control. These results suggest that the ethanolic left extract of *cc* could have neuroprotective properties, which could be attributed to its antioxidant properties.

## 1. Introduction

Cognitive disorders refer to the deterioration of intellectuals and faculties, including memory, orientation, concentration, attention, learning capacity, judgment, and language [1]. All neuropsychological disorders that lead to partial or complete memory loss are included in amnesia [2]. Amnesia occurs when learning and short- or long-term storage of information are disrupted [2] and describes the pathological inability to learn new information or to remember information already acquired. In 2015, the Alzheimer's Disease International report on dementia estimates that about 46.8 million people in the world are affected by dementia. More than half (58%) of the affected population resides in low- and middle-income countries [3].

Despite the effectiveness of benzodiazepine therapy to alleviate anxiety and depressive symptoms, many patients refuse or discontinue treatment because of adverse effects such as memory loss, dependence, sedation, and muscle relaxation [4]. Memory is a cognitive aspect very sensitive to benzodiazepines, which influence the acquisition phase [5]. Diazepam (DZP), one of the most widely clinically used benzodiazepines, has been administrated for the induction of amnesia. This is due to its strong ability to potentiate the effects of GABA by binding to one of the allosteric regulatory sites of its receptor. The resulting increase in the opening frequency of chloride channels leads to hyperpolarization, making the cell more difficult to be excited [6]. In the literature, DZP is known to be used as a model of induction of amnesia [7]. Acute administration of diazepam may cause anterograde amnesia [8]. According to Georgieva-Kotetarova et al. Reference [9], an intraperitoneal injection of 2.5 mg/kg of DZP induces amnesia in rodents. In the brain, the hippocampus, located in the temporal lobe, has a major role in learning and memory. It is a plastic and vulnerable structure that gets damaged by a variety of stimuli [10] (Anand and Dhikav, 2012). In a patient called Henry Gustav Molaison (called HM), the removal of the hippocampus due to refractory epilepsy induce anterograde and partial retrograde amnesia [11] (Preilowski, 2009). Many studies have shown different conditions that affect the hippocampus and produce changes ranging from molecules to morphology [10] (Anand and Dhikav, 2012).

To manage amnesia, conventional treatment consists of taking drugs such as donepezil, rivastigmine, galantamine, and memantine which have the ability to reduce the amnesic effect and electroshocks in mice [10], but these treatments still have adverse effects in our patients. Alternative therapies consisting of plant-derived medications are increasingly being used to relieve neurodegenerative disorders [12]. In most developing countries, the vast majority (80%) of the population utilize traditional medicine for their primary health care [13]. Many plants were used in the treatment of amnesia such as *Daniellia oliveri* [14], *Thespesia populnea* [15], and *Rumex vesicarius* [16].

*Crassocephalum crepidioides* (Asteracaeae) is an annual herb native to tropical Africa and Madagascar. This plant is used for the treatment of headaches and epilepsy in Cameroon and Nigeria [17]. Documented evidence of pharmacological activity revealed antiinflammatory, immunomodulatory, antigenotoxic, and antidiabetic activities of *Crassocephalum crepidioides* leaf extract [18]. The phytochemical screening of Crassocephalum crepidioides recorded the presence of substances such as tannins, coumarins, combined anthracene derivatives C-heterosides, flavonoids, mucilage, reducing compounds, and Steroids [17]. It is known that the flavonoids contain in this plant exert a multiplicity of neuroprotective actions within the brain, including a potential to protect neurons against injury induced by neurotoxins, an ability to suppress neuroinflammation, and the potential to promote memory, learning, and cognitive function [19]. However, the neuroprotective effect of this plant has not been investigated. In this study, we evaluated the neuroprotective effects of ethanolic leaf extract of *cc* on diazepam-induced amnesian mice.

## 2. Materials and Methods

### 2.1. Plant Material and Extraction

*Crassocephalum crepidioides* leaves were harvested in Fongo-Ndeng (West region of Cameroon) and identified by Pr. Tchobsala, Botanist at the University of Maroua. The plant was then authenticated at the National Herbarium of Cameroon in comparison to a voucher specimen (reference number 24250/SRF Cam). After drying, the leaves were ground to obtain a powder. The ethanolic extract was prepared by soaking 500 g of this powder in 3 L of ethanol 95%. The mixture was macerated for 72 hours, then filtered using a Whattman No. 4 filter paper. The filtrate was evaporated under reduced pressure using a rotary evaporator (BUCHI brand rotavapor, R.300), resulting in 32 g of extract (yield 6,4%).

### 2.2. Chemicals

Diazepam, Piracetam, ketamine, and ethanol 95% were purchased from SigmaAldrich, USA. All drugs and extracts were freshly prepared in ethanol 95% on the day of the experiment.

### 2.3. Animal Material

Male Swiss mice aged between 8 and 12 weeks and weighing 25–30 g were purchased from the National Veterinary Laboratory (LANAVET) in Garoua - Cameroon. Before the beginning of the experiment, animals were acclimatized for 2 weeks at the Laboratory of the Department of Biological Sciences, Faculty of Sciences, University of Maroua. They were housed in a controlled environment (room temperature: around 25°C; natural illumination: approx. 12 h light/dark cycle). Animals had free access to food and tap water *ad libitum*. Animal handling and experiments were carried out in accordance with the guideline of the Cameroonian Bioethics committee (Reg N° FWA-IRB00001945) and following the HIN-care and use of laboratory animals manual (8^th^ Edition).

### 2.4. Experimental Design

Thirty mice distributed into six groups of five mice each were used. The Normal control and Negative control groups received 2% ethanol *per os*, the positive control group received piracetam (150 mg/kg, *p. o*), and three experimental groups were treated with three doses of ethanolic leaf extract of *Cc* (100, 200 and 400 mg/kg, *p. o*) respectively. All groups except the Normal control group were co-treated with Diazepam (3 mg/kg, *i. p*) daily for 14 days (Table 1). The volume of administration of each substance was 10 mL/kg. To evaluate the beneficial effects of the extract on memory, the Radial Arm Maze (RAM) and the Novel Object Recognition (NOR) tests were used, while the antidepressive effect was evaluated by using the tail suspension test (TST). Each behavioral test was performed thirty minutes after the administration of diazepam. At the end of behavioral studies, animals were sacrificed under diazepam/ketamine (10 mg/kg and 50 mg/kg, *i. p*., respectively) anesthesia, their brains were harvested and pathologies were assessed on the left hemisphere using H&E staining. Hippocampi, isolated from the right hemisphere, were used to prepare homogenates for the determination of oxidative stress biomarkers: superoxide dismutase (SOD) and malondialdehyde (MDA).

### 2.5. Behavioral Tests

#### 2.5.1. Radial Arm Maze Test

The RAM test is used in the laboratory to evaluate memory in rodents. The device consisted of 8 arms, numbered 1 to 8 (48 × 12 cm) extending from a central cylindrical platform (32.5 cm in diameter). Arms (1, 3, 5, and 7) were baited and this device was raised 50 cm above the floor. Based on food reinforcement, this test was carried out in two phases: a habituation phase (5 to 7 days) and a test phase (7 days). Each animal had to learn to visit the baited arms without returning to them during the same test (working memory). It also had to learn to avoid entering unbaited arms (reference memory). Mice had to use the knowledge acquired during habituation to consume the food hidden at the end of the baited arms. A working memory error was recorded if the animal returns to a baited arm that it was previously visited, while a reference memory error was recorded if it visits the unbaited arms. The session ended either when all baited arms were visited or when five minutes had elapsed. After each passage, the device was cleaned with 70% ethanol, to eliminate as much as possible, the residual odors left by the previous mouse [20].

### 2.6. The Novel Object Recognition Test

The NOR test is used in the laboratory to evaluate memory in rodents. The open field arena maze with object recognition for long-term memory was used as previously described by of El-Marasy et al. [21]. Briefly, the test was achieved in 3 phases (habituation, acquisition, and retention over three days):

- During habituation, each mouse was placed at the center of the arena and allowed to explore the arena for 5 minutes to familiarize itself with the experimental set-up
- The next day, during the acquisition phase, each mouse was subjected to a test (E1), in which two identical objects or familiar objects (F) were placed in the arena at opposite corners
- Twenty-four hours after E1, the retention phase was carried out (E2). A new object (N) replaced one of the familiar objects and the mouse was exposed this time to two different objects.

Exploration was considered when the animal directed its nose towards the object (2 cm away from it). After each passage, the device was cleaned with 70% ethanol, to eliminate as much as possible, the residual odors left by the previous mouse. The distinction between the familiar object (F) and the new object (N) at E2 was measured by comparing the time spent exploring the familiar object (F) with the time spent exploring the new object (N).

## 3. Antidepressant Test

### 3.1. Tail Suspension Test

The TST was used to assess depressive behavior. The hemodynamic constraint of being suspended uncontrollably by the tail forces the animal to engage in three types of escape behavior: forward or backward running; twisting of the body with attempts to catch the suspended; temporary body shaking followed by episodes of immobility [22].

The mouse was hung up by the tail and hooked with duct tape for 6 minutes. The total duration of immobility was calculated when the strength of the mouse movements was below a predefined threshold. The optimal lower threshold is determined by comparing the immobility results with the results of the automated device. The stillness was considered when there was an absence of initiated movements and passive swaying. An upper threshold is also determined to detect only vigorous movements.

#### 3.1.1. Variables Measured

- The immobility time: a period when there is an absence of initiated movements and passive swaying, jerking of the body, temporally followed by episodes of immobility
- The mobility time: a period when the animal exercises vigorous movements such as movements of running forward or backward and twisting movements

## 4. Biochemical Assays of Oxidative Stress Biomarkers

### 4.1. Determination of Malondialdehyde Concentration

The level of lipid peroxides was evaluated by the method described by Ohkawa et al. [23]. Briefly, 200 mL of supernatant collected from hippocampus homogenate was mixed with 1 mL of 50% trichloroacetic acid in 0.1 M HCl and 1 mL of 26 mM thiobarbituric acid. After vortex mixing, the sample was maintained at 95°C for 20 min. Furthermore, the samples were centrifuged at 9609 rpm for 10 min and the supernatants absorbances were read at 532 nm. A calibration curve was constructed using MDA as standard and the results were expressed as *μ*mol/g of the organ.

### 4.2. Determination of Superoxide Dismutase Activity

#### 4.2.1. Principle

The presence of SOD in the sample inhibits the oxidation of adrenaline to adrenochronoma. The increase in absorbance, which is proportional to SOD activity, is noted between 20 and 80 seconds at 480 nm [24]. Different solutions were prepared: Carbonate buffer (0.05 M; pH = 10.2) by dissolving 4.5 g of sodium carbonate (NaCO_3_; 10 H_2_O) and 4.2 g of monosodium carbonate (NaCO_3_) in 500 mL of distilled water. The pH of the solution was adjusted to 10.2 with 1 M sodium hydroxide and the volume made up to 1000 mL with distilled water. The 0.06 mg/mL epinephrine solution was prepared in the dark, by dissolving 6 mg of adrenaline in distilled water to a final volume of 100 mL.

## 5. Histological Analysis

Histological analyses of brain (hippocampi) were assessed from 5 *μ*m sections of paraffin-embedded tissues. Coronal sections were made from the brain (left hemisphere) in the hippocampus region using the Mouse Brain Atlas with the following coordinates (anterior/posterior *D* 2.0 mm, medial/lateral *D* 1.5 mm, and dorsal/ventral AP *D* 2.0 mm) [25]. Following hematoxylin-eosin staining, the brain section was assessed on microphotographs using a digital camera attached to a light microscope (Scientico, Haryana, India).

### 5.1. Statistical Analysis

All results were expressed as mean ± S. E. M. For the RAM and NOR tests, data were analyzed by two-way; ANOVA (NOR Radial Maze) followed by Bonferroni and Tukey post hoc tests, respectively. All analyses were performed using Graph Pad Prism software (version 8.0.1., San Diego, California, USA). Results were considered significant at *p* < 0.05.

## 6. Results

### 6.1. Effects of the Ethanolic Leaf Extract of *Crassocephalum crepidioides* on Memory Evaluated by the Radial Arm Maze

**Figure 1:**
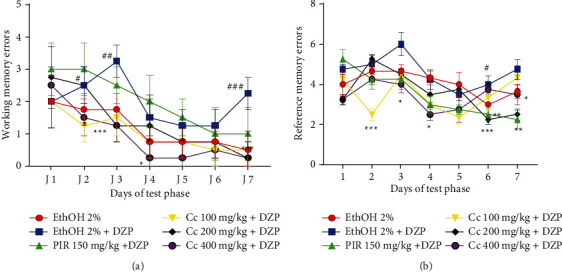
Effect of ethanolic extract of *Cc* on the working (a) and reference (b) memory errors of mice in the radial maze test. Each point represents the mean ± ESM, *n* = 5. DZP = diazepam, PIR = piracetam, Cc = ethanolic extract of *Crassocephalum crepidioides*. ^*∗*^*p* < 0.05, ^*∗∗*^*p* < 0.01, and ^*∗∗∗*^*p* < 0.001 vs. negative control group. ^*#*^*p* < 0.05, ^*##*^*p* < 0.01, and ^*###*^*p* < 0001 vs. normal control group.

The analysis of Figure 1 showed that, compared to the normal control group, Diazepam-group injection solely scored significantly increased (*p* < 0.05, *p* < 0.01, *p* < 0001) number of working (Figure 1(a)) and reference (Figure 1(b)) memory errors. The different doses of ethanolic leaf extract of Cc significantly decreased the number of working memory errors, especially on day 14 (day 7 of the RAM test phase) compared to the negative control group (Figure 1(a)). Treatment with the plant extract at all tested doses also induced a significant decrease (*p* < 0.05, *p* < 0.01, and *p* < 0001) of the number of reference memory errors compared to the negative control group (Figure 1(b)). The extract induces similar effects to the positive control group that received the piracetam (150 mg/kg) used as a reference drug.

### 6.2. Effects of the Ethanolic Leaf Extract of *Cc* in Memory Evaluated by the Novel Object Recognition Test

**Figure 2:**
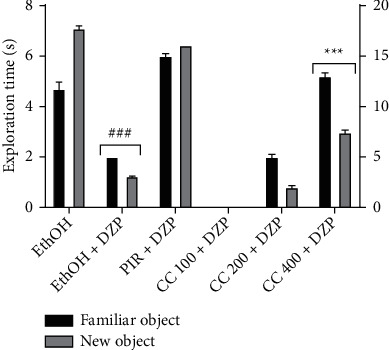
Effects of ethanolic leaf extract of *Cc* in memory evaluated by the Novel Object Recognition test. Bars represent the mean ± SEM; n = 5. DZP = diazepam, PIR = piracetam, Cc = ethanolic extract of *Crassocephalum crepidioides*. ^*∗∗∗*^*p* < 0.001 vs. negative control group. ^*###*^*p* < 0001 vs. normal control group.

As shown in Figure 2, animals treated with diazepam only showed a significant decrease (*p* < 0001) of time spend to explore the novel object in comparison to the normal control group. Compared to the negative control group, animals treated with the extract at the dose of 400 mg/kg as well as the positive control group, induced a significant (*p* < 0001) increase in this exploration time.

### 6.3. Antidepressive Effects of the Ethanolic Leaf Extract of *Crassocephalum crepidioides* Evaluated by the Tail Suspension Test

**Figure 3:**
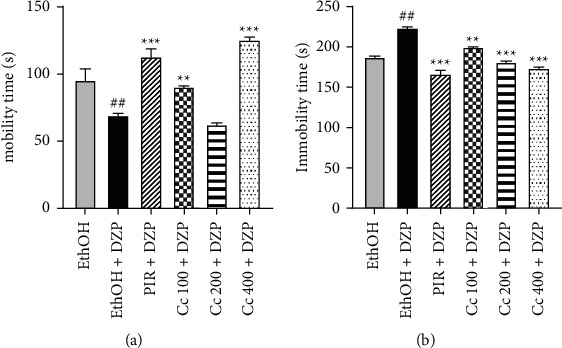
Effects of ethanolic extract of *Crassocephalum crepidioides* on mobility (a) and immobility (b) time of in mice in the tail suspension test. Bars represent the mean ± SEM; *n* = 5. DZP = diazepam, PIR = piracetam, Cc = ethanolic extract of *Crassocephalum crepidioides*. ^*∗∗*^*p* < 0.01 and ^*∗∗∗*^*p* < 0.001 vs. negative control group. ^*##*^*p* < 0.01 vs. normal control group.

Figure 3 shows the antidepressant effects of ethanolic leaf extract of *C* in TST. Compared to the normal control group, the animals of the negative control group showed a decrease in mobility time and a significant (*p* < 0.05) increase of immobility time. As well as the Positive control group, the pretreatment of mice with the *Cc* extract induced a significant (*p* < 0.01 and *p* < 0001) (Figure 3(a)) increase of the mobility time and a significant (*p* < 0001) decrease of immobility time in comparison to the negative control group (Figure 3(b)).

### 6.4. Effects of the Ethanolic Leaf Extract of *Crassocephalum crepidioides* on Oxidative Stress Parameters

**Figure 4:**
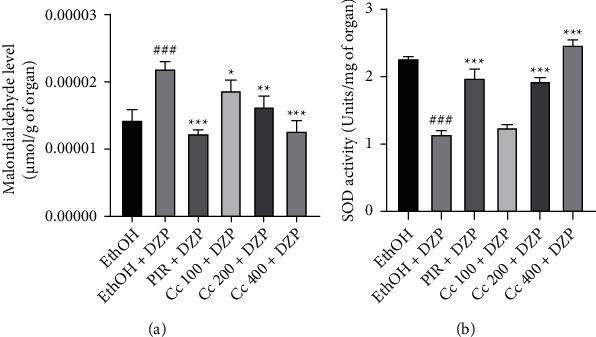
Effect of ethanolic extract of *Cc* on the malondialdehyde level (a) and superoxide dismutase level (b) in the hippocampus of mice. Bars represent the mean ± SEM; *n* = 5. DZP = diazepam, PIR = piracetam, Cc = *Crassocephalum crepidioides*. ^*∗*^*p* < 0.05, ^*∗∗*^*p* < 0.01, and ^*∗∗∗*^*p* < 0.001 vs. negative control group. ^*###*^*p* < 0001 vs. normal control group.

The analysis of Figure 4 showed that DZP caused a significant increase (*p* < 0001) in MDA (Figure 4(a)) levels and a significant (*p* < 0001) decrease of SOD activity (Figure 4(b)) in the hippocampi of mice of the Negative control group compared to the Normal control group. As well as piracetam, the treatment with the ethanolic extract of *Cc* reversed the effects induced by diazepam. The treatment of mice with the extract at all tested doses induced a significant decrease (*p* < 0.05, *p* < 0.01, *p* < 0001) of MDA levels (Figure 4(a)) and a significant (*p* < 0001) increase of SOD activity (Figure 4(b)) in comparison to the Negative control group.

### 6.5. Effect of the Ethanolic Leaf Extract of *Crassocephalum crepidioides* on Hippocampi Microarchitecture

**Figure 5:**
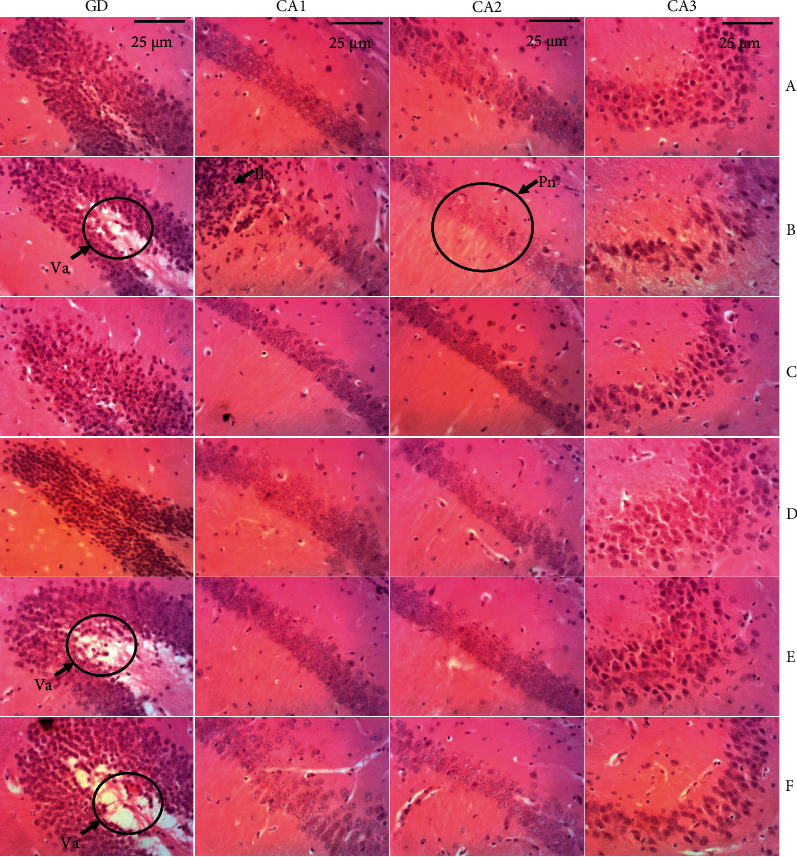
Effect of the ethanolic extract of *Cc* on the microarchitecture of the hippocampus Photomicrographs of the hematoxylin-eosin stain of the Dente Gyrus (×100) and Cornus Ammonis 1, 2, and 3 (X200); scale bar = 25 *μ*m. *A* = normal control, *B* = negative control (DZP), *C *= positive control (piracetam + DZP), *D* = CA 400 mg/kg + DZP, *E* = CA 200 mg/kg + DZP, *F* = CA 100 mg/kg + DZP, GD = Dente Gyrus, CA (1, 2, and 3) = Cornus Ammonis (regions 1, 2 and 3), Va = neuronal vacuolation, Il = leukocyte infiltration, and Pn = neuronal loss.

Figure 5 has shown the effect of administration of the ethanolic extract of *Cc* on the microarchitecture of the hippocampus. The hippocampi sections of mice of normal control (*A*) group presented a normal structure with neurons appearing intact in the different hippocampi regions (DG, CA1, CA2, and CA3). Compared with the normal control group, the sections of the Negative control group (B) displayed several histopathological changes in the hippocampus, marked by neuronal vacuolation (DG region), leukocyte infiltration (CA1 region), and neuronal loss (CA1, CA2, and CA3 regions) (Figure 5). As well as Piracetam (C), the treatment of mice with the extract of *Cc* prevents the adverse effects induced by Diazepam (Figure 5). The best effects of the extract treatment were obtained at the dose of 400 mg/kg (*D*).

## 7. Discussion

To investigate the neuroprotective effect of *Cc* on diazepam-induced amnesia, we used behavioral experiments such as the RAM test to evaluate long-term and short-term memory; NOR tests to evaluate long-term memory, and TST to evaluate depression. Benzodiazepines are a class of drugs that impair memory consolidation by interfering in information transfer from short-term memory to long-term memory [5], providing an additional rationale for conducting these experiments. Many patients taking benzodiazepines for the treatment of various neurological disorders often suffer from amnesia as a side effect [4].

The RAM is a very suitable tool for evaluating the action of drugs on memory [9], including both working memory (which is a form of short-term memory) and reference memory (long-term memory) in rodents [26]. Thus, treatment with the ethanolic left extract of *Cc* (100, 200, and 400 mg/kg), resulted in a significant reduction in the number of errors in reference and working memories in comparison to the negative control group. According to some authors, the extract that induces such an effect in RAM tests are able to improve memory [26, 27]. This reduction in the number of errors in reference and working memory could be linked to the action of the flavonoids and alkaloids present in this extract [25]. The beneficial effect of the *Cc* to improve memory was confirmed by the Test.

The NOR test measures the natural ability of a rodent to explore a new object compared to a familiar object [28]. Results of NOR tests are influenced by the integrity of specific regions of the brain: the hippocampus and the cortex [29]. The present data, therefore, show that diazepam would alter the memory process at the level of the two brain regions. In this study, compared to the negative control group, animals treated with the *Cc* extract at the dose of 400 mg/kg induced a significant increase in the exploration time of the new objects. The preference for the new object in the animals treated with the extract makes it possible to conclude an improvement in the cognitive properties of the drug and, in particular, its beneficial effect in improving memory [30]. The novel object preference of animals treated with the extract at the dose of 400 mg/kg provides information on baseline memory in these mice: this extract could protect brain against neurodegeneration in the hippocampus and cortex. The effects induced by the extract suggest that the secondary metabolites contained in this extract would have neuroprotective effects on the two regions of the brain aroused.

Acute administration of DZP in adult rats induces depression of functional brain activity, but in a more limited number [31]. The results obtained in this study make it possible to obtain information on the role that certain cerebral structures may have in the various pharmacological effects of DZP. The significant decrease in the mobility time and increase in the time of immobility of the negative control group compared with the normal control observed during the TST would reveal depressive-type behavior due to diazepam. Compared to the negative control group, the treatment of mice with the *Cc* extract at the doses of 200 and 400 mg/kg induced a significant increase in the mobility time. The same extract also induced a significant decrease in the immobility time of the mice at the dose of 400 mg/kg compared to the negative control group. These effects induced by the *Cc* extract in this test could justify the antidepressant properties of this extract. These properties could be attributed to the presence of flavonoids and tannins in the extract [17]. Indeed, memory loss (short-term memory loss) could be linked to depression. Compounds such as flavonoids and tannins present in this plant extract with their antidepressant properties could explain the beneficial effects of this plant on memory. Flavonoids are a large class of secondary metabolites found in plants and in various foods. Many flavonoids possess antioxidant and antidepressant activities. It has been reported that the neuroprotective mechanisms of the antidepressant effects of flavonoids remain unclear. This is because it is proposed that flavonoids generally exert their antidepressant effects by altering behavior, cytokine levels, oxidative stress and energy metabolism parameters. In addition to antioxidant action, each flavonoid follows one or more different pathways to act against depression [32]. Tannins, polyphenolic compounds, are biological molecules with many pharmacological properties, including neuroprotection [33]. Tannins (tannic acid) are described as a potent antidepressant due to their activity in reducing neurodegeneration and inhibiting monoamine oxidase [34].

The results of behavioral tests generally reflect the complex biochemical processes that occur at the level of the central nervous system, more precisely at the level of the hippocampus and the prefrontal cortex. The hippocampus is a mid-temporal lobe structure involved in declarative memory in humans and spatial memory in rodents [35]. Brain cells are known to contain a very high percentage of long-chain polyunsaturated fatty acids. Reactive Oxygen Species (ROS) are continuously generated in the nervous system during normal metabolism and normal neuronal activity. The brain is regularly subject to free radical-induced lipid peroxidation. It is also known that the protective system in the brain is poor against oxidative stress, compared to other tissues [30]. Malondialdehyde results from the direct attack of vulnerable amino acid side chains by free radicals [35]. In this study, mice in the negative control group exhibited a significant increase in the MDA level and a significant decrease in the activity of SOD in comparison to the normal control group. These results are in line with reports which revealed that the administration of diazepam in rodents can lead to increase brain oxidative stress [36]. Furthermore, the administration of different doses of the ethanolic extract of *Cc* resulted in a decrease in MDA level and a significant increase in the activity of SOD. These effects of the extract on oxidative stress parameters could be due to flavonoid compounds founds in this plant. It is well known that flavonoids are powerful antioxidants that act by suppressing the formation of EROS, either by enzymatic inhibition or by chelation of trace elements involved in the generation of free radicals; either by trapping ROS and/or increasing the regulation of antioxidants [27]. Thus, we can suggest that the ethanolic extract of *Cc* is endowed with antioxidant properties. The neuroprotective effect of the ethanolic extract of *Cc* could be explained by its ability to inhibit lipid peroxidation and neutralize ROS in the hippocampus.

The hippocampus mediates several higher brain functions, such as learning, memory, and spatial coding [37]. Located in the temporal lobe, it is one of the oldest parts of the brain and is part of the limbic system. The hippocampus is composed of the dentate gyrus and the Cornu Ammonis (CA). The dentate gyrus contains the fascia dentata and hilum (CA4), while the CA is differentiated into distinct regions: CA1, CA2, and CA3 [38]. The dentate gyrus is the input region of the hippocampus, and it plays a critical role in learning, memory, and spatial coding processes. It acts as a preprocessor of incoming information, preparing it for further processing in CA3 [37]. Both hippocampal areas CA1 and CA3 contribute to context-dependent extinction acquisition, but only CA1 is required for contextual memory retrieval. Research in the dorsal CA1 and dorsal CA3 subregions of the hippocampus has shown that these regions play an important role in mediating temporal order memory for spatial location information. However, only the dorsal CA1 region is essential for processing temporal information about visual objects without affecting the detection of novel visual objects [39]. The CA2 region of the hippocampus is a somewhat obscure area whose form and function are not understood. Until recently, the CA2 region was considered simply an extension of the CA3 region, with some referring to it as the CA3a region. One process that has been shown to depend on CA2 is the ability to recognize a novel or familiar conspecific, known as social recognition memory [40]. The CA3 region has a specific role in memory processes, seizure susceptibility, and neurodegeneration. Recurrent axonal collaterals of CA3 pyramidal cells branch widely making excitatory contacts with neighboring excitatory and inhibitory neurons. This circuit is involved in the encoding of spatial representations and episodic memories [38]. The histological analysis of brain sections showed neuronal vacuolation (DG region), leukocyte infiltration (CA1 region), and neuronal loss (CA1, CA2, and CA3 regions) in the hippocampi of the negative control group in comparison to the Normal control group. This deterioration of brain architecture induced by DZP in the negative control group could justify the memory loss and depression observed in the same animals in this study. Animals treated with the extract at the dose of 400 mg/kg presented and architecture similar to those of the normal control group: no neuronal degeneration was noted. Any disturbance (degeneration) of a neuron at the level of the hippocampus leads to difficulty or inability to learn [41] (Williams and Herrup, 2001). The ethanolic extract of the leaves of *Cc* at a dose of 400 mg/kg would therefore protect neurons against the lesions induced by diazepam. The ethanolic extract of the leaves of *Cc* could therefore produce its effects either by acting as an antagonist of DZP receptors, or by inhibiting lipid peroxidation as it increases antioxidant activity.

## 8. Conclusion

This study aimed to evaluate the neuroprotective effects of the ethanolic leaf extract of *Cc* on diazepam-induced amnesia in mice. The results obtained strongly showed that the ethanolic leaf extract of *Cc* (100, 200, and 400 mg kg) effectively protected memory processes from the diazepam-induced damage in mice. It emerges that this extract has effects that prove to be neuroprotective against the disorders induced by diazepam. More precisely, this extract could have improved long-term and short-term memory; it could have antioxidant and antidepressant properties and could have protected the hippocampus from the neurotoxic effect of diazepam. These beneficial effects of the *Cc* extract could justify the use of this extract to manage nervous system diseases in African traditional medicine.

## Figures and Tables

**Table 1:** Categorization of mice and dosage of drugs in each group.

Groups	Treatments	Doses
1: normal control	2% ethanol (EthOH)	//
2: negative control	2% EthOH + diazepam (DZP)	3 mg/kg
3: positive control	Piracetam (PIR) + DZP	150 mg/kg
4: test 1	Extract + DZP	100 mg/kg
5: test 2	Extract + DZP	200 mg/kg
6: test 3	Extract + DZP	400 mg/kg

## Data Availability

The data used in this study can be obtained from the corresponding author upon reasonable request.
